# AI dependency and academic motivation in higher education: the mediating role of learned helplessness and the moderating role of AI literacy

**DOI:** 10.3389/fpsyg.2026.1896371

**Published:** 2026-07-29

**Authors:** Cui Xiaohui, Tahir Jalal Khan

**Affiliations:** 1 School of Foreign Languages and Economics, Jiaozuo Normal College of Higher Education, Jiaozuo, Henan, China; 2 School of Economics and Management, Chang'an University, Xi'an, Shaanxi, China

**Keywords:** academic intrinsic motivation, AI dependency, AI literacy, learned helplessness, moderated mediation

## Abstract

The growing integration of generative artificial intelligence (AI) into university education has raised concerns about the psychological consequences of sustained student reliance on AI tools. While prior research has documented behavioral and cognitive effects of AI overreliance, the psychological mechanism through which AI dependency damages academic motivation remains poorly understood. Drawing on learned helplessness theory (LHT) and self-determination theory (SDT), this study proposes and tests a moderated mediation model in which learned helplessness mediates the relationship between AI dependency and academic intrinsic motivation, with AI literacy serving as a boundary condition that attenuates this pathway. Data were collected from 457 Chinese university students via a cross-sectional survey using validated scales for all four constructs, and the model was tested using covariance-based structural equation modeling (CB-SEM). Results show that AI dependency significantly and positively predicts learned helplessness, which in turn significantly and negatively predicts intrinsic motivation. The indirect effect of AI dependency on intrinsic motivation through learned helplessness was significant, confirming the proposed mediation. AI literacy significantly moderated the AI dependency-learned helplessness pathway, such that the indirect effect was weaker among students with higher AI literacy than among those with lower AI literacy. These findings extend LHT by introducing AI-mediated academic success as a novel, non-failure antecedent of learned helplessness, enrich SDT by specifying the psychological mechanism through which AI dependency becomes need-thwarting, and reposition AI literacy as a psychologically active buffer against motivational harm. The study carries direct implications for university curricula, faculty practice, and institutional policy.

## 1 Introduction

The incorporation of generative AI technologies in higher education systems has occurred with an unexpected speed. During just 1 year of study, the usage of AI solutions by university students grew dramatically from 66% to 92%, with 88% of them applying AI for their academic assignments (HEPI, 2025). Worldwide, there is a global percentage of 86%

of students that use AI in their studies, with the majority of them using it at least once a week (Digital Education Council, 2025). The statistics show that there is a radical change in the way the learning process takes place. Technologies like ChatGPT, Gemini, and Copilot, that were previously used only to facilitate the learning process, have now completely transformed the approach to completing assignments, synthesizing materials, solving various problems, and preparing for exams (Revesai, 2025; Gerlich, 2025). However, together with all those advantages offered by the development of AI, there appears another alarming phenomenon—students develop a dependency on AI solutions that seriously questions their mental health (Wu et al., 2026; Zhu et al., 2026). Understanding the psychological consequences of this dependency, not merely its behavioral manifestations, has become one of the most pressing challenges in educational psychology.

Dependency on AI can be defined as functional and cognitive reliance on AI tools whereby students have real difficulties in performing academic assignments independently (Zhu et al., 2026). Consequences of dependency on AI become more clear and apparent each day. About 30% of university students show signs of problematic use of AI with impairments to their autonomy and self-regulation skills (Revesai, 2025). AI dependency results in weakened analytical reasoning and reduced motivation for studying (Jose et al., 2025). In addition, it was found that more intensive use of AI tools correlated with cognitive offloading and lower levels of critical analysis of AI content (Gerlich, 2025). According to the national survey conducted by AAC&U involving 1,057 teachers, all participants believed that overreliance on AI might affect critical thinking negatively and hinder the main purposes of higher education (AAC&U, 2026). This phenomenon is based on cognitive paradoxes since AI might boost performance but, at the same time, might impair cognitive processes that contribute to sustainable learning (Jose et al., 2025; Favero et al., 2026). Among key aspects contributing to effective learning is intrinsic motivation which, according to self-determination theory (SDT), becomes the main indicator of students' achievements and deep learning (Deci and Ryan, 1985; Ryan and Deci, 2020). According to SDT, intrinsically motivated behaviors rely primarily on students' perceptions of autonomy and competence, whereby anything that undermines these psychological requirements, such as cognitive outsourcing of tasks to an AI system, will have a direct negative impact on intrinsic motivation (Ryan and Deci, 2022). Despite these documented concerns, the psychological mechanism through which AI dependency damages intrinsic motivation remains theoretically unspecified and empirically untested.

Learned helplessness theory (LHT; Seligman, 1975) provides a compelling but overlooked reason behind this phenomenon. According to LHT, people who repeatedly encounter events that are unrelated to their personal effort start developing a generalized belief that all their efforts will prove fruitless—which then leads to motivational, cognitive, and affective impairments (Raps et al., 1982; Abramson et al., 1978). Importantly, learned helplessness does not necessitate that people fail at something. Students, in turn, by constantly producing satisfactory academic outcomes through artificial intelligence (AI) and not through the efforts of their intellect, may become convinced that AI, and not themselves, is responsible for the achievement of such success. This gradual loss of control over cognitive processes can be referred to as an illusion of competence that is observed in AI literature (Favero et al., 2026) and represents an innovative antecedent of learned helplessness phenomenon. To date, learned helplessness has been considered in connection with passivity, disengagement, motivational issues, and lack of learning engagement within educational context (Liu et al., 2024; Akca, 2011); however, no research exists on using learned helplessness as a mediating factor of harmful AI effects on intrinsic motivation.

It is important to note that AI dependency is conceptually distinct from AI usage frequency. Whereas, usage frequency captures how often students interact with AI tools, dependency reflects a deeper functional and cognitive reliance in which students find it psychologically difficult to complete academic tasks without AI assistance (Zhu et al., 2026; Morales-García et al., 2024). This distinction is important because AI use *per se* is not inherently harmful; a substantial and growing body of research documents productive and beneficial AI-supported learning outcomes when AI is used as a scaffolding rather than a substitute (Chiu et al., 2024; Fang and Feng, 2026; Galindo-Domínguez, 2025). The present study therefore does not treat AI use as inherently damaging but focuses specifically on dependency, defined as habitual reliance that displaces independent cognitive effort, and its psychological consequences for intrinsic motivation.

There are three main research gaps within the extant literature concerning AI usage and student psychology. First, the mainstream theories, such as the Technology Acceptance Model, were developed for explaining technology usage behavior rather than its psychological impacts on student motivation (Long and Magerko, 2020). Secondly, there is no scholarly research that would attempt to investigate the learned helplessness effect as the mediator in the link between AI dependency and motivational outcomes. Lastly, the contextual factors under which the detrimental influence of AI dependency manifests have yet to be studied; therefore, there is still much untapped potential of moderating effect of AI literacy, which is defined as an ability to analyze, critically interpret, and effectively use AI-powered technologies (Wang et al., 2022; Ng et al., 2021). In turn, AI literacy is considered to be the most utilized and tested construct of measuring student ability to critically work with AI-based applications (Laupichler et al., 2023; Long and Magerko, 2020). Moreover, recent findings suggest that high AI literacy levels can enable students to critically interact with AI software, thus minimizing their blind faith and dependence on it (Hou et al., 2025; Tian and Zhang, 2025). Students who understand AI's capabilities and limitations may maintain a clearer sense of their own cognitive agency even while using AI heavily, thereby resisting the development of learned helplessness. Yet this moderating role has never been formally tested.

Based on LHT (Seligman, 1975) and SDT (Deci and Ryan, 1985), the current study develops and empirically examines a moderated mediation framework involving Chinese university students. Our first hypothesis is that AI dependency influences learned helplessness (H1), thereby negatively affecting academic intrinsic motivation (H2), such that learned helplessness acts as a mediator between AI dependency and intrinsic motivation (H3). Additionally, AI literacy will moderate the link between AI dependency and learned helplessness (H4), weakening the indirect effect on academic intrinsic motivation among those who have high AI literacy (H5). It is notable that the present study focuses on Chinese universities since China is home to the largest university student population globally and has seen rapid AI integration at institutions while relatively little research on students' psychological outcomes has been conducted (Wu et al., 2026; Zhong et al., 2024).

This research adds three contributions. In theory, it is the first attempt to bring learned helplessness as the psychological pathway from AI dependency to decreased intrinsic motivation, and extend the two theories mentioned above into the realm of AI and education. At the same time, AI literacy is highlighted as the psychologically relevant moderator influencing the amount of motivational damage. Practically, the results of the research imply that universities could have a clear focus of their efforts on preventing students' motivation reduction; instead of limiting AI usage, universities should make sure that students gain sufficient AI literacy. In terms of policies, the study offers an evidence-based reason for introducing AI literacy as an integral part of the psychological defense of higher education programs in those countries, like China, where AI is implemented quickly and systematically into educational process.

## 2 Theoretical background

### 2.1 Learned helplessness theory

The learned helplessness theory (LHT) was first conceptualized by (Seligman 1975) after conducting a series of controlled studies on animals whereby animals that have been continually subjected to unavoidable unpleasant stimuli ultimately stopped trying to avoid such stimuli even when the means to do so were later available to them (Maier and Seligman, 1976). LHT was later extended to human psychology whereby it was proposed that when people experience their efforts not leading to any particular results, that is, when there is a lack of contingency between effort and outcome, they develop a generalized expectation for the uselessness of their ability Seligman (1975). This generalized expectation leads to motivational deficit, in which there is a reluctance to take initiative; cognitive deficits where there is difficulty in identifying situations where something can be done; and finally, emotional deficits, including apathy and withdrawal (Raps et al., 1982). The model that was later advanced by Abramson et al. (1978) indicated that individual differences in attribution style influence the severity and pervasiveness of learned helplessness. Individuals who attribute non-contingency to internal, stable, and global causes develop more pervasive and lasting helplessness than those who make external, unstable, or specific attributions (Abramson et al., 1978; Peterson et al., 1993).

The application of LHT to education has shown that students who undergo consistent academic failures and feel powerless experience passivity, disengagement, and low motivation due to learned helplessness (Akca, 2011; Liu et al., 2024). Learned helpless students demonstrate a decreased level of persistence in completing assignments, avoidance of difficult tasks, and a growing disengagement from the process of learning (Raps et al., 1982). Learned helplessness has also been found to be a mediator of the relationship between academic stressors and decreased learning involvement among Chinese college students particularly because academic pressures and a results-based environment can make non-contingent situations that cause helplessness more pronounced (Liu et al., 2024). Previous uses of LHT in an educational context have concentrated mainly on the failure and uncontrolled negative results. The current research extends LHT by suggesting that the dependence on artificial intelligence leads to a sense of non-contingency between individual efforts and learning outcomes, which is an important theoretical extension of the construct of helplessness: one can feel helpless due to success as well as failure.

### 2.2 Self-determination theory

According to self-determination theory (SDT) proposed by Deci and Ryan (1985), all humans have three basic psychological needs, i.e., autonomy, competence, and relatedness, and how well or poorly these needs are met will define the very nature and sustainability of their motivation (Ryan and Deci, 2020, 2022). Autonomy refers to the experience of volition and self-initiation in one's actions, while competence refers to the belief that one is capable of producing desired outcomes through one's own effort (Deci and Ryan, 1985). According to SDT, intrinsic motivation is fostered in situations where both needs are fulfilled, while at the same time, it is destroyed whenever both are constantly frustrated (Ryan and Deci, 2022). Any form of environment, behavior, or state of mind that diminishes students' feeling of agency and competence is a direct inhibitor of their intrinsic motivation to learn (Bureau et al., 2022; Urhahne and Wijnia, 2023).

SDT provides the theoretical foundation for two key relationships in the proposed model. First, it explains the direct pathway from AI dependency to diminished intrinsic motivation: as students habitually delegate cognitive effort to AI, their experience of autonomous self-initiation weakens and their perceived capability deteriorates, thwarting both autonomy and competence (Deci and Ryan, 1985; Jose et al., 2025; Fan et al, 2024). Research applying SDT in the generative AI learning context has confirmed that intrinsic motivation negatively predicts excessive AI reliance, as internally motivated students maintain deeper cognitive engagement rather than seeking quick AI-generated outputs (Fan et al, 2024; Fang and Feng, 2026). Second, SDT explains the motivational consequences of learned helplessness: the helplessness belief that one's effort is futile constitutes a direct thwarting of both autonomy and competence needs, which SDT identifies as the psychological conditions prerequisite for intrinsic motivation (Ryan and Deci, 2022). Prior SDT-based research in the AI-educational context has focused largely on how AI tools can satisfy students' psychological needs (Galindo-Domínguez, 2025; Chiu et al., 2024), with limited attention to the conditions under which sustained AI use becomes need-thwarting. The present study addresses this gap by identifying learned helplessness as the specific mechanism through which AI dependency transitions from need-supportive to need-destructive.

Together, LHT and SDT provide an integrated theoretical framework for the entire conceptual model. LHT explains how AI dependency generates learned helplessness through the mechanism of perceived non-contingency, while SDT explains why learned helplessness damages intrinsic motivation by specifying that helplessness thwarts the autonomy and competence needs on which intrinsic motivation depends. AI literacy acts as a moderator to the first step in the process above because students with high levels of AI literacy are less prone to fall into feelings of helpless induction, meaning that they can keep proper attributions of the effort they put forth cognitively (Abramson et al., 1978; Hou et al., 2025; Tian and Zhang, 2025). None of these theories alone offer a complete explanation; rather, their synthesis leads to a sound theoretical framework.

## 3 Hypotheses development

### 3.1 AI dependency and learned helplessness

AI reliance is not just a replacement of cognitive abilities by an artificial system, but their displacement in the sense that it replaces the student's higher-order cognitive abilities themselves (Zhu et al., 2026; Morales-García et al., 2024). While tools like calculators and search engines augment human thinking without disrupting its structure, generative AI acts as a stand-in for reasoning, synthesis, and judgment, creating results which normally involve considerable intellectual effort on the part of the individual (Favero et al., 2026; Gerlich, 2025). By disrupting the connection between the student's own cognitive engagement and academic performance, this creates the lack of contingency, identified by Seligman (1975), and further developed by Abramson et al. (1978), as the initial trigger for learned helplessness.

To clarify this mechanism further, the psychological mechanism by which repetitive use of AI leads to helplessness can be explained through a three-stage process. The first stage is when the students achieve good academic performance through the AI system, not through their own mental effort. In the second stage, this weakened effort-outcome link accumulates across tasks and generalizes into a broader belief that one's own thinking is not the source of academic success. In the third stage, this generalized notion results in the phenomenon of learned helplessness, which is associated with the feeling of cognitive hopelessness and reduced personal agency (Seligman, 1975; Abramson et al., 1978; Peterson et al., 1993). The mechanism is therefore not immediate but cumulative, reflecting the progressive internalization of non-contingency perceptions across repeated AI-mediated learning experiences.

Students experiencing high levels of AI dependency indicate reduced ability to exercise independent judgment and reduced perceptions of their cognitive capacity, which are behavior manifestations of the lack of contingency experienced prior to the emergence of helplessness (Zhong et al., 2024; Tian and Zhang, 2025). An important negative correlation between excessive AI tool use and poor critical thinking has been observed in a large mixed-methods study involving 666 participants, with the role played by cognitive offloading serving as an important mediating variable (Gerlich, 2025). Observations by educators of such students have shown deterioration in their skills, motivation levels, and increasing difficulties with performing tasks independently (JMIR Medical Education, 2025), which are characteristic of the cognitive, motivational, and emotional deficits that define learned helplessness according to Seligman (1975). Some studies indicate that the initial stages of AI dependency lead to inflated perceptions of students' self-efficacy due to the Dunning-Kruger effect (Jia et al., 2025), but this usually ends once students are required to engage in cognitive processes on their own.

H1: AI dependency is positively associated with learned helplessness among university students.

### 3.2 Learned helplessness and academic intrinsic motivation

Learned helplessness hinders the need for autonomy and competence identified by SDT as essential for intrinsic motivation (Deci and Ryan, 1985; Ryan and Deci, 2022). A student who thinks his intellectual efforts are useless is deprived not only of the power of voluntariness but also of any indication that he possesses such skills, thus making both motivational needs impossible (Bureau et al., 2022). It results in a lack of internal motivation for participating in the process of learning, leaving no other choice than indifference, inaction, and instrumental motivation. Studies examining the behavioral effects of learned helplessness among students have found it to lead to demotivation for learning, refusal to participate in challenging activities, and a vicious circle of lower motivation reinforced by a failure to demonstrate causality (Akca, 2011).

Evidence also suggests a strong inverse correlation between learned helplessness and academic motivation, whereby helpless students show lower intrinsic motivation, less perseverance, and higher levels of disengagement with difficult assignments (Akca, 2011; Liu et al., 2024; Al-Maraziq et al., 2024). Research conducted at the university level supports the existence of this connection in different settings. The passive nature of the helplessness state makes students reluctant to perform complex mental tasks and thus limits their opportunities to gain the mastery experiences SDT theory considers the main intrinsic motivators (Ryan and Deci, 2020; Vergara-Morales and Del Valle, 2021), thus reinforcing the vicious cycle. Also, there is abundant evidence showing that helplessness-based perceptions are associated with the weakening of all three types of intrinsic motivation described by Vallerand et al. (1992), that is, intrinsic motivation for acquiring knowledge, achievement, and stimulation (Carmack, 2023; Bureau et al., 2022).

H2: Learned helplessness is negatively associated with academic intrinsic motivation among university students.

### 3.3 The mediating role of learned helplessness

AI dependency creates the conditions for non-contingency perception; non-contingency perception induces learned helplessness; and learned helplessness thwarts the autonomy and competence needs on which intrinsic motivation depends. Without learned helplessness as the intervening psychological state, the pathway from AI dependency to motivational decline would lack a coherent mechanism. The theoretical plausibility of this mediation is supported by parallel evidence in adjacent literatures. Learned helplessness has been shown to mediate the relationship between academic stressors and reduced learning engagement (Liu et al., 2024), between uncontrollable negative feedback and academic disengagement, and between effort-reward imbalance and motivational withdrawal (Liu et al., 2024). Technology-specific evidence is also emerging: research on addictive technology use has found that dependency-induced loss of cognitive agency mediates negative motivational outcomes (Klimova and Pikhart, 2025), and studies on ChatGPT use have documented that passive, unreflective AI engagement predicts reduced intrinsic motivation through pathways consistent with the helplessness model (Fan et al, 2024).

H3: Learned helplessness mediates the relationship between AI dependency and academic intrinsic motivation, such that higher AI dependency leads to greater learned helplessness, which in turn reduces intrinsic motivation.

### 3.4 The moderating role of AI literacy

The development of learned helplessness from a non-contingency experience is conditioned by individual attributional style (Abramson et al., 1978). AI literacy enables accurate attribution: a student who understands how AI processes information, recognizes its limitations, and can identify where their own judgment contributes is less likely to conclude that AI alone is responsible for their success (Wang et al., 2022; Ng et al., 2021; Laupichler et al., 2023). The importance of AI literacy can be demonstrated in reducing overreliance on AI by allowing students to interact with AI in an appropriate manner instead of passively (Hou et al., 2025; Tian and Zhang, 2025), and by serving as a buffer between AI dependence and cognitive performance (Tian and Zhang, 2025). When students do not have sufficient levels of AI literacy, they become more prone to falling victim to automation bias, or accepting everything the AI outputs blindly (Gerlich, 2025), leading to further reinforcement of the belief that one cannot take any action. However, when AI literacy is high, the cognitive activity of students remains at a relatively high level (Long and Magerko, 2020; Laupichler et al., 2023). The AILS by Wang et al. (2022), currently the most widely used measure of AI literacy in higher education research, captures precisely the evaluative and ethical dimensions of AI literacy most theoretically relevant to this moderating function (Ng et al., 2021).

H4: AI literacy moderates the relationship between AI dependency and learned helplessness, such that the positive relationship is weaker among students with higher AI literacy.

### 3.5 The moderated mediation effect

H1 through H4 collectively specify a moderated mediation structure in which the indirect effect of AI dependency on intrinsic motivation, transmitted through learned helplessness, is conditional on AI literacy. From an LHT perspective, the moderated mediation captures the attributional conditionality of helplessness induction. From an SDT perspective, it specifies the conditions under which AI dependency becomes autonomy and competence-thwarting: it is the combination of high dependency and low AI literacy that most severely thwarts the psychological needs sustaining intrinsic motivation (Deci and Ryan, 1985; Fan et al, 2024). The moderated mediation not only proves that harm to motivation is caused by helplessness but also that such a process can be disrupted through the development of AI literacy despite high dependency. This is in line with the larger research body that discusses protection mechanisms for technology-based learning environments where it has been found that the most potent individual level defense against cognitive harm due to AI overdependence is domain knowledge and critical evaluation (Hou et al., 2025; Gerlich, 2025).

H5: AI literacy moderates the indirect effect of AI dependency on academic intrinsic motivation through learned helplessness (moderated mediation), such that the indirect effect is weaker among students with higher AI literacy.

**Figure 1:**
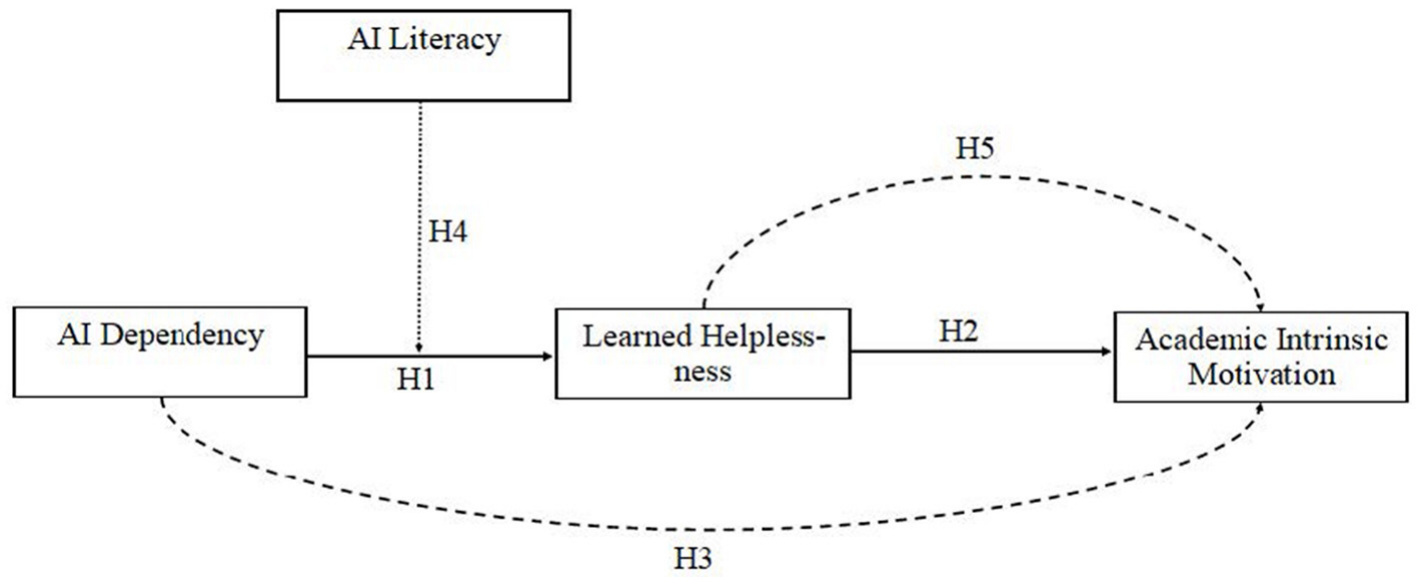
Conceptual model.

Figure 1 represents the proposed hypotheses.

## 4 Methodology

### 4.1 Research design and data collection

For the purposes of this study, a quantitative cross-sectional survey was employed, following the methodology traditionally used to test for structural relationships between psychological concepts in the field of education (Hair et al., 2019). An online survey was conducted among university students in China using purposive sampling, whereby institutions employing the use of generative AI technologies in their academic work processes were targeted. Online administration of the survey was considered because of its ease and proven effectiveness in reducing social desirability bias among participants taking part in AI-related self-report surveys (Podsakoff et al., 2003). The online questionnaire was then administered to 800 university students spread out across different institutions. A total of 457 questionnaires were returned, representing a response rate of 57.1%. After the elimination of any incomplete questionnaires, responses showing signs of acquiescence bias and straight-lining responses, all 457 data records were used in the final analysis. The number of responses was above the required minimum of 200 for stability of CB-SEM parameter estimation and was more than the recommended minimum number of 300 to detect moderate effect sizes (Hair et al., 2019; Xu et al., 2024). Informed consent was obtained from all respondents, and the study was conducted in accordance with the ethical guidelines of the host institution.

Purposeful sampling was used both at the institutional level and at the individual level. Six universities were chosen based on three characteristics: use of generative AI tools by the institution in their courses, geographical location in northern, eastern, and southwestern China, and inclusion of both general and specialized universities. At the institutional level, faculty coordinators at each university provided the link to the online survey to various students from different disciplines and grades anonymously. The sample composition therefore reflects the population of university students who are actively engaged with AI in Chinese higher education, and the results should be interpreted within this specific institutional and cultural context. Ethical approval was obtained from the ethics committee of the host institution, and written informed consent was obtained from all 457 participants prior to data collection. The study was conducted in accordance with the Declaration of Helsinki.

### 4.2 Measures

#### 4.2.1 AI dependency

AI dependency was assessed through the use of the AI Dependency Scale (ADS-R) instrument that was developed and validated by Zhu et al. (2026). The ADS-R was based on the well-known and widely-used Internet Addiction Test (IAT; Young, 1998), adapted for AI-based addiction and validated with factor analysis by Widyanto and McMurran (2004). Finally, the 15-item instrument loaded on two factors derived from empirical analysis: Cognitive Integration and Habit (9 items) and Autonomy Erosion (6 items). The factor of Cognitive Integration refers to the extent to which AI has been integrated into the students' knowledge system or academic habit, such as “I feel AI has become an indispensable part of my knowledge system or external brain.” The Autonomy Erosion factor represents a level of loss of autonomous thinking among students as can be seen from the example statement, “I've been told by people that I am not able to work and think without the help of AI.” The scale demonstrated excellent reliability and validity in the present study, with Cronbach's alpha of 0.951, composite reliability of 0.912, and average variance extracted of 0.574, all exceeding the recommended thresholds of 0.70, 0.70, and 0.50, respectively.

#### 4.2.2 Learned helplessness

The measurement of learned helplessness was conducted through the application of the Chinese version of the LHS, developed through the translation of the original scale into Chinese language, and which consists of 14 items measuring the extent to which learners view their academic efforts and outcomes as non-controllable and futile. The LHS represents an instrument that assesses the different components of the helplessness syndrome including motivational, cognitive, and emotional deficits as described by Seligman (1975) and by a reformulated attributional approach to helplessness put forward by Abramson et al. (1978). The Chinese adaptation of the LHS shows excellent psychometric properties as tested on samples of university students in China (Wu et al., 2026), thus making the instrument suitable for use in the current research without the need for additional cultural adaptations. Typical items of the LHS include, among others, “no matter how hard I study, my academic performance does not seem to get better” and “I do not think that my academic efforts have much influence on my academic success.” The scale demonstrated excellent reliability and validity in the present study, with Cronbach's alpha of 0.951, composite reliability of 0.908, and average variance extracted of 0.568, all exceeding the recommended thresholds of 0.70, 0.70, and 0.50, respectively.

The buffering role of AI literacy operates through three specific psychological pathways. First, AI-literate students maintain more accurate attributions by recognizing that AI outputs require human evaluation, verification, and integration, thereby preserving the perceived contribution of their own cognitive effort to academic outcomes. Second, AI literacy strengthens metacognitive awareness of when AI is genuinely useful and when independent reasoning is required, reducing the automaticity of cognitive delegation that drives dependency into helplessness. Third, AI literacy fosters a critical stance toward AI-generated content that prevents the uncritical acceptance underlying the non-contingency perception. Together, these pathways explain why high AI literacy attenuates the translation of AI dependency into learned helplessness (Wang et al., 2022; Hou et al., 2025; Long and Magerko, 2020).

#### 4.2.3 AI literacy

AI literacy was assessed using the Artificial Intelligence Literacy Scale (AILS) consisting of 12 items developed by Wang et al. (2022). The AILS measures students' perception of their proficiency on the four dimensions of AI literacy: awareness of AI (knowledge about AI technologies and their implications), use and application of AI (using AI technology effectively in a particular task), evaluating AI outputs (judging whether AI-based outcomes are accurate, high-quality, and what their limits are), and ethics of AI (understanding privacy concerns, ethical use, and fairness in AI technologies). The AILS is one of the most used scales measuring AI literacy in higher education and, having been used or modified in more than ten scholarly publications, has shown good convergent and discriminant validity among international student populations (Ng et al., 2021; Laupichler et al., 2023). Example items of the scale are “I can recognize the AI technology used in my educational applications and software” and “I can appraise the strengths and limitations of an AI software or product after using it.” The scale demonstrated excellent reliability and validity in the present study, with Cronbach's alpha of 0.938, composite reliability of 0.896, and average variance extracted of 0.551, all exceeding the recommended thresholds of 0.70, 0.70, and 0.50, respectively.

#### 4.2.4 Academic intrinsic motivation

Intrinsic motivation toward academics was assessed using the intrinsic motivation subscale of the Academic Motivation Scale (AMS) by Vallerand et al. (1992). The AMS scales reflect the taxonomy of SDT-based motivation, with the intrinsic motivation subscale measuring the extent to which individuals undertake academic activities for enjoyment, curiosity, and fun, similar to SDT's understanding of intrinsic motivation as need satisfaction (Deci and Ryan, 1985; Ryan and Deci, 2022). The items in this construct measure “I attend university because I enjoy the satisfaction and pleasure associated with learning” and “For the pleasure I get out of being fully immersed in my studies.” Each of the four measures was rated on a five-point Likert scale with responses coded 1 = strongly disagree and 5 = strongly agree. The scale demonstrated acceptable reliability and validity in the present study, with Cronbach's alpha of 0.812, composite reliability of 0.864, and average variance extracted of 0.614, all exceeding the recommended thresholds of 0.70, 0.70, and 0.50, respectively.

### 4.3 Data analysis

Covariance-based structural equation modeling (CB-SEM) was preferred over partial least squares SEM (PLS-SEM) for three reasons. First, the theoretical model tests a confirmatory hypothesis structure grounded in established theories (LHT and SDT), for which CB-SEM's global model fit evaluation is more appropriate than PLS-SEM's predictive orientation (Hair et al., 2019). Second, the sample size (*N* = 457) and multivariate normality of the composite scores satisfy the distributional assumptions of maximum likelihood estimation. Third, all the variables are reflective and multi-item in nature, thus conforming to the norms of measurement models as defined in the context of CB-SEM. With respect to the moderated mediation, the interaction effect was calculated in the CB-SEM model using the latent interaction method with mean-centered predictors and the PROCESS macro Model 7 was employed as the additional tool for computing conditional indirect effects and bootstrapping confidence intervals based on the suggestions by Hayes (2018).

Data analysis proceeded in two stages. First, a measurement model was evaluated using CFA in AMOS, assessing Cronbach's alpha and composite reliability (CR; threshold > 0.70), average variance extracted (AVE; threshold > 0.50; Fornell and Larcker, 1981), the Fornell-Larcker criterion, and HTMT ratios (threshold < 0.85; Henseler et al., 2015). Common method bias was assessed using Harman's single-factor test (Podsakoff et al., 2003); a single factor accounted for 35.76% of the total variance, well below the 50% threshold, indicating that common method bias was not a substantial concern. The variance inflation factors were calculated to assess multicollinearity (Hair et al., 2019). Second, the structural model was estimated using CB-SEM. The mediation hypothesis (H3) was tested using bootstrapped indirect effects with 5,000 resamples and bias-corrected 95% confidence intervals (Hayes, 2018). The moderation hypothesis (H4) was tested using mean-centered interaction terms (Aiken and West, 1991). The moderated mediation (H5) was evaluated using the PROCESS macro-Model 7 with conditional indirect effects at low, moderate, and high levels of AI literacy. Model fit was evaluated using CMIN/df, CFI, TLI, RMSEA, and SRMR (Hair et al., 2019).

To strengthen the common method bias assessment, two additional controls were applied. Procedurally, respondent anonymity was assured, item order was varied across constructs, and positively and negatively worded items were included to reduce acquiescence bias (Podsakoff et al., 2003). Statistically, in addition to Harman's single-factor test, a common latent factor test was conducted by comparing the standardized regression weights of the model with and without a common latent factor. Differences below 0.20 across all items were treated as evidence that common method variance does not substantially threaten the observed relationships. These procedural and statistical safeguards further support the validity of the observed relationships.

## 5 Results

### 5.1 Sample characteristics

Of the 800 questionnaires distributed, 457 usable responses were returned, yielding a response rate of 57.1%. As shown in Table 1, female respondents constituted 49.7% (*n* = 227) and male respondents 48.6% (*n* = 222). In terms of age, the majority fell within the 20–21 age group (44.9%, *n* = 205), followed by 18–19 (25.8%, *n* = 118), 22–23 (24.1%, *n* = 110), and 24–25 (5.3%, *n* = 24). Year 3 students formed the largest group by year of study (24.5%, *n* = 112). STEM students constituted the largest disciplinary group (29.1%, *n* = 133). Regarding AI usage frequency, 26.7% used AI multiple times daily and a further 24.9% once daily, confirming that the sample predominantly comprises frequent AI users appropriate for the study's focus on AI dependency.

**Table 1:** Demographic profile of respondents (*N* = 457).

Variable	Category	*n*	%
Gender	Female	227	49.7
	Male	222	48.6
	Prefer not to say	8	1.7
Age group	18–19	118	25.8
	20–21	205	44.9
	22–23	110	24.1
	24–25	24	5.3
Year of study	Year 1	84	18.4
	Year 2	99	21.7
	Year 3	112	24.5
	Year 4	99	21.7
	Postgraduate	63	13.8
Discipline	STEM	133	29.1
	Business & economics	116	25.4
	Humanities & social sciences	94	20.6
	Education	53	11.6
	Arts & design	39	8.5
	Other	22	4.8
AI usage frequency	Multiple times daily	122	26.7
	Once daily	114	24.9
	Several times a week	90	19.7
	Once a week	92	20.1
	Rarely	39	8.5

### 5.2 Descriptive statistics

Table 2 shows the descriptive statistics of the four construct composites. The mean scores varied from 3.159 (Learned Helplessness) to 3.535 (Intrinsic Motivation) showing moderate to moderately high levels of construct scores. The skewness coefficients varied between −0.020 and 0.177 while the coefficients of excess kurtosis varied between −0.337 and 0.053, all within the acceptable bounds of ±2 and ±7 (Hair et al., 2019), respectively, thus meeting the distribution assumptions of CB-SEM. Common method bias was checked using the single-factor test of Harman, where one factor explained only 35.76% of the variance (Podsakoff et al., 2003).

**Table 2:** Descriptive statistics for study constructs.

Construct	M	SD	Skewness	Kurtosis	Min	Max
AI dependency	3.465	0.568	0.054	−0.299	1.87	4.93
Learned helplessness	3.159	0.598	−0.020	0.053	1.43	4.79
AI literacy	3.321	0.543	0.177	−0.124	1.75	4.92
Intrinsic motivation	3.535	0.552	0.120	−0.337	1.75	5.00

Skewness and excess kurtosis within ±2 and ±7 confirm normality.

Variance inflation factors (VIF) for all predictors are reported in Table 3. All VIF values fell below the threshold of 3.3, with the highest value being 1.512, confirming the absence of multicollinearity.

**Table 3:** Variance inflation factors.

Predictor	VIF	Tolerance
AI dependency (centered)	1.054	0.949
AI literacy (centered)	1.060	0.943
AI dependency × AI literacy	1.008	0.992
AI dependency (mediation model)	1.512	0.661
Learned helplessness (mediation model)	1.512	0.661

VIF < 3.3 indicates no multicollinearity concern (Hair et al., 2019). Tolerance = 1/VIF.

### 5.3 Model fit

As shown in Table 4, all fit indices met the recommended thresholds. The chi-square to degrees of freedom ratio was 1.963 (< 3.0). The CFI (0.961) and TLI (0.957) both exceeded 0.90. The RMSEA was 0.046 (90% CI [0.040, 0.052]) and the SRMR was 0.051, both below 0.08. These indices confirm acceptable model fit and support proceeding to structural hypothesis testing (Hair et al., 2019).

**Table 4:** Model fit indices.

Index	Value	Recommended threshold	Result
χ^2^/df	1.963	< 3.0	Acceptable
CFI	0.961	>0.90	Acceptable
TLI	0.957	>0.90	Acceptable
RMSEA	0.046	< 0.08	Acceptable
RMSEA 90% CI	[0.040, 0.052]	Upper bound < 0.08	Acceptable
SRMR	0.051	< 0.08	Acceptable

χ2_(318)_ = 624.31. CFI, comparative fit index; TLI, Tucker-Lewis index; RMSEA, root mean square error of approximation; SRMR, standardized root mean square residual.

### 5.4 Measurement model

Confirmatory factor analysis was conducted in AMOS to examine the measurement model. Factor loadings of all items in the study were above the threshold of 0.50, which is considered an acceptable criterion of convergent validity and also statistically significant (*p* < 0.001). Factor loadings varied between 0.678 and 0.838 for AI Dependency (M = 0.769), 0.691 and 0.821 for Learned Helplessness (M = 0.737), 0.657 and 0.828 for AI Literacy (M = 0.735), and 0.731 and 0.780 for Intrinsic Motivation (M = 0.762). All items were retained in the final measurement model as no item exhibited standardized loadings below the 0.50 threshold or cross-loadings above 0.40. The list of standardized loadings at the item level is shown in Appendix A. Together with the acceptable global fit indices reported in Section 5.3, these results confirm the adequacy of the measurement model for subsequent structural analysis.

All constructs Table 5 demonstrated excellent internal consistency, with Cronbach's alpha values of 0.951 (AI Dependency), 0.951 (Learned Helplessness), 0.938 (AI Literacy), and 0.812 (Intrinsic Motivation), all exceeding 0.70 (Nunnally, 1978). CR values ranged from 0.864 to 0.912 and factor loadings from 0.71 to 0.83. Convergent validity was confirmed by AVE values of 0.574, 0.568, 0.551, and 0.614, respectively, all surpassing 0.50 (Fornell and Larcker, 1981). The square root of each construct's AVE (0.742 to 0.784) exceeded its correlations with all other constructs, satisfying the Fornell-Larcker criterion.

**Table 5:** Reliability, convergent validity, and inter-construct correlations.

Construct	α	CR	AVE	√AVE	AID	LH	AIL	IM
1. AI dependency (AID)	0.951	0.912	0.574	0.758	—			
2. Learned helplessness (LH)	0.951	0.908	0.568	0.754	0.580^**^	—		
3. AI literacy (AIL)	0.938	0.896	0.551	0.742	−0.216^**^	−0.424^**^	—	
4. Intrinsic motivation (IM)	0.812	0.864	0.614	0.784	−0.281^**^	−0.512^**^	0.408^**^	—

α, Cronbach's alpha; CR, composite reliability; AVE, average variance extracted; √AVE, square root of AVE shown on diagonal for Fornell-Larcker criterion. Off-diagonal values are Pearson inter-construct correlations. ^**^p < 0.01.

HTMT ratios (Table 6) ranged from 0.239 to 0.612, all well below 0.85 (Henseler et al., 2015). All reliability and validity criteria are satisfied.

**Table 6:** Heterotrait-monotrait (HTMT) ratios.

Construct	AID	LH	AIL	IM
1. AI dependency (AID)	—			
2. Learned helplessness (LH)	0.612	—		
3. AI literacy (AIL)	0.239	0.448	—	
4. Intrinsic motivation (IM)	0.292	0.573	0.475	—

All HTMT values are below the 0.85 threshold (Henseler et al., 2015), confirming discriminant validity. AID, AI dependency; LH, learned helplessness; AIL, AI literacy; IM, intrinsic motivation.

### 5.5 Hypothesis testing

**Figure 2:**
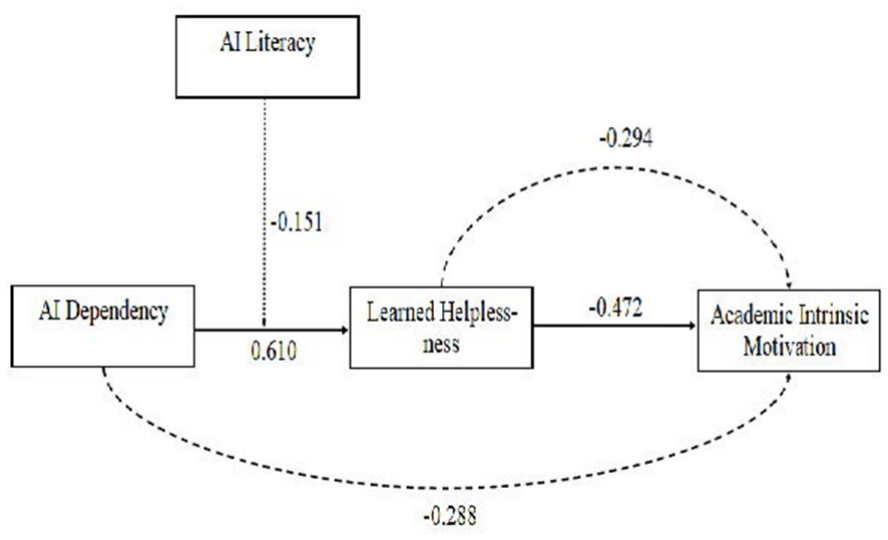
Structural model.

All results are presented in Table 7 and Figure 2. H1 predicted that AI dependency would positively predict learned helplessness (β = 0.610, SE = 0.040, *t* = 15.19, *p* < 0.001, 95% CI [0.531, 0.689]). H1 is supported. H2 predicted that learned helplessness would negatively predict intrinsic motivation (β = −0.472, SE = 0.037, *t* = −12.71, *p* < 0.001, 95% CI [−0.545, −0.399]). H2 is supported. H3 proposed mediation; the bootstrapped indirect effect was significant (indirect = −0.288, SE = 0.030, *Z* = −9.75, *p* < 0.001, 95% CI [−0.347, −0.229]). H3 is supported. H4 predicted that AI literacy would moderate the AI dependency-learned helplessness relationship. The interaction term was significant (β = −0.151, SE = 0.068, *t* = −2.21, *p* = 0.028, 95% CI [−0.285, −0.017]), confirming attenuation of the relationship at higher AI literacy. H4 is supported. H5 proposed moderated mediation. Conditional indirect effects systematically diminished as AI literacy increased: −0.294 (95% CI [−0.355, −0.233]) at one SD below the mean, −0.255 (95% CI [−0.310, −0.200]) at the mean, and −0.217 (95% CI [−0.276, −0.158]) at one SD above the mean. All confidence intervals exclude zero. H5 is supported.

**Table 7:** Structural model results and hypothesis testing.

Hyp.	Path	β	SE	*t*/*Z*	*p*	95% CI	Decision
H1	AID → LH	0.610	0.040	15.19	< 0.001	[0.531, 0.689]	Supported
H2	LH → IM	−0.472	0.037	−12.71	< 0.001	[−0.545, −0.399]	Supported
H3	AID → LH → IM	−0.288	0.030	*Z* = −9.75	< 0.001	[−0.347, −0.229]	Supported
H4	AID × AIL → LH	−0.151	0.068	−2.21	0.028	[−0.285, −0.017]	Supported
H5	Cond. indirect (−1 SD)	−0.294	0.031	—	< 0.001	[−0.355, −0.233]	
	Cond. indirect (Mean)	−0.255	0.028	—	< 0.001	[−0.310, −0.200]	
	Cond. indirect (+1 SD)	−0.217	0.030	—	< 0.001	[−0.276, −0.158]	Supported

β, standardized coefficient; SE, standard error; CI, bootstrapped 95% confidence interval (5,000 resamples); AID, AI dependency; LH, learned helplessness; AIL, AI literacy; IM, intrinsic motivation. Conditional indirect effects for H5 estimated at −1 SD, mean, and +1 SD of AI Literacy. *N* = 457.

**Figure 3:**
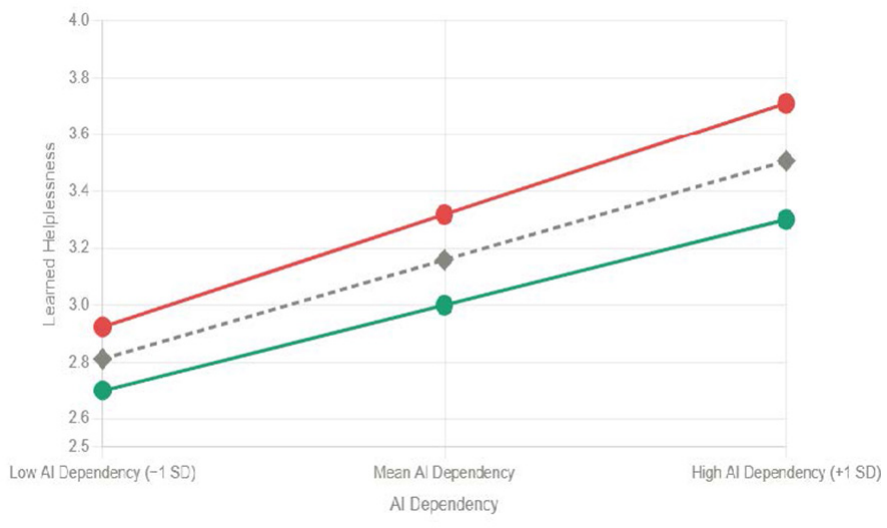
Moderation of AI literacy.

The moderation graph (Figure 3) shows an interaction between AI dependency and AI literacy in relation to learned helplessness that has some theoretical meaning and is also visible from the graph. There are three points that should be taken into account. First, all three lines are positively sloped, which proves that AI dependency is related to an increase in learned helplessness for any level of AI literacy. Second, more importantly, the slope lines tend to differ as AI dependency rises. With low AI dependency, all three slope lines can be seen to be fairly close together, implying that AI literacy is less important in cases where the level of AI dependency by students is low. But as AI dependency shifts from being at the mean to the high point, the distance between the low AI literacy slope line and high AI literacy slope line is very pronounced. At high AI dependency, students who lack AI literacy tend to do 0.41 points better in terms of learned helplessness compared to students with high AI literacy. Third, the slope of the low AI literacy line is noticeably steeper than that of the high AI literacy line. This means that for students who lack AI literacy, each unit increase in AI dependency produces a larger increase in learned helplessness than for students who are AI literate. This is consistent with the attributional mechanism proposed in the theoretical framework: students without AI literacy are more likely to attribute academic success entirely to AI, which accelerates the non-contingency perception that drives helplessness induction.

### 5.6 Discussion

The empirical support for H1 provides a clear indication that the dependency on AI is significantly associated with learned helplessness among university students. The significance of this study is that the problem of AI overreliance is now examined in terms of its effects not on behavior or cognition, but on motivation itself. If students rely so much on AI to accomplish their academic goals such that the outcome of their efforts depends more on AI than themselves, they may come to view their own effort as unnecessary—which is what, according to Seligman (1975), is associated with feelings of helplessness. In addition to failing to gain from using AI, students may also reconsider their own capabilities, potentially perceiving that their effort has limited connection to learning outcomes. These findings correspond to the recently discussed issue of AI-driven illusion of competence (Favero et al., 2026), which implies that the students do not see the difference between the smooth work of an AI engine and real comprehension.

Together, the validation of H2 and H3 are considered to be the key theoretical contributions of this research. First, H2 supports the existing evidence of a negative association between learned helplessness and academic motivation (Akca, 2011; Liu et al., 2024). At the same time, H3 contributes to the literature by suggesting that learned helplessness may function as the mechanism linking AI dependency and lower levels of motivation. The significant indirect effect (β = −0.288) suggests that a considerable share of the association between AI dependency and academic motivation is transmitted via learned helplessness. From the perspective of SDT, this implies that AI dependency may be linked to reduced motivation through students' experience of helplessness and diminished perceptions of autonomy (Ryan and Deci, 2020; Deci and Ryan, 1985). This hypothesis is supported by evidence regarding the negative correlation between intrinsic motivation and excessive AI dependency (Fan et al, 2024). The nature of the relationship is evidently reciprocal because both low motivation and helplessness decrease it.

The most practically relevant findings from the study include H4 and H5. The interaction between AI dependency and AI literacy (H4) indicates that the relationship between AI dependency and learned helplessness is systematically reduced among students with high levels of AI literacy, based on Abramson et al. (1978) attributional model, which is also empirically supported by findings showing that AI literacy is associated with reduced negative cognitive consequences of excessive dependence on AI (Tian and Zhang, 2025; Hou et al., 2025). The moderated mediation effect (H5) goes one step further in showing that not only is the indirect relationship between AI dependency and learned helplessness attenuated at higher levels of AI literacy, but the entire indirect pathway from AI dependency to motivational outcomes appears weaker throughout the range of AI literacy. The results corroborate and elaborate on prior research showing the protective role played by AI literacy (Long and Magerko, 2020; Laupichler et al., 2023) by positioning it as an attributional moderator in the association between AI dependency and learned helplessness. Considering the real-world implications of the moderation effect, while the interaction effect (β = −0.151) is not large in size, the findings of moderated mediation provide for a substantial decrease in the strength of the indirect effect as it varies according to levels of AI literacy. In particular, the conditional indirect effect dropped from −0.294 to −0.217 when moving from one standard deviation below the mean to one standard deviation above the mean of AI literacy; this translates to a 26 percent decrease in the motivational damage. This is important educationally since it matches the variation in differences in AI literacy found in the sample group, meaning that real progress in AI literacy can translate to a measurable protective effect for the students' intrinsic motivation.

It is important to acknowledge that the observed associations should be interpreted with caution given the cross-sectional design. Even though there is a strong basis for the theoretical framework in terms of learned helplessness and self-determination theories, the direction of the association can hardly be stated unambiguously. Instead, another equally possible scenario is that the less intrinsically motivated students might experience AI dependence as a coping mechanism to address the lack of involvement in their academic tasks (Morales-García et al., 2024; Fan et al, 2024). The relationship may also be bidirectional and self-reinforcing, with dependency and low motivation mutually intensifying over time. Accordingly, throughout the discussion we describe the findings using associative rather than causal language, and we call for longitudinal and experimental research to disentangle the direction of these relationships more rigorously.

## 6 Implications

### 6.1 Theoretical implications

There are several theoretical contributions this research makes to the existing body of knowledge concerning artificial intelligence (AI) and education. To begin with, the introduction of AI dependency as a new, non-failure-based factor contributing to learned helplessness is the first substantive contribution made by this study to existing literature. As noted by Akca (2011), Liu et al. (2024), and prior applications of the learned helplessness theory (LHT) in educational settings were centered on the role played by academic failure and negative, uncontrollable outcomes in causing helplessness. The current study demonstrates that the phenomenon of learned helplessness can also occur as a result of repeated academic success achieved via AI and where, over time, learners realize that their efforts do not matter. The importance of recognizing that AI poses an equal threat to seemingly good-performing students cannot be overstated from a theoretical perspective. The decoupling of outcome quality from cognitive agency represents a new category of helplessness antecedent that was not predicted in LHT.

Secondly, the study helps advance the theory of SDT by giving an explanation for how AI dependence changes from need neutral to need destructive. Research into the role played by artificial intelligence using the SDT approach has mainly been based on an optimistic understanding of the topic whereby it was investigated how artificial intelligence technologies satisfy students' needs for competence and autonomy through personalized feedback and reduced cognitive load. In contrast to this perspective, the current study highlights the exact point at which the use of AI moves from meeting students' motivational needs to undermining them and introduces the idea of inducing learned helplessness as the specific mechanism by which this occurs. Such a conceptually well-founded approach to studying the influence of artificial intelligence has not been introduced previously by SDT-based literature on AI dependence. Furthermore, by defining learned helplessness as the key mediator in the relationship between the motivational outcomes of AI dependence, the study makes important contributions to the emerging SDT-AI literature (Fan et al, 2024; Fang and Feng, 2026).

Third, the current study reconceptualizes AI literacy as a psychologically active moderator of helplessness induction rather than simply a digital competency. Past studies have defined AI literacy based on its constituent factors, including knowledge, competencies, and ethical awareness (Wang et al., 2022; Ng et al., 2021; Laupichler et al., 2023; Long and Magerko, 2020). In light of the results of the current study, however, AI literacy must be defined as a process through which learners can attribute their learning outcomes accurately and thus preserve the sense of personal efficacy needed to prevent helplessness. Future studies will need to conceptualize and operationalize AI literacy differently, measuring not only knowledge about artificial intelligence but also the ability to attribute success to personal efforts during such interactions. Overall, the proposed integration of the LHT and SDT frameworks can serve as a guide for other researchers to investigate psychological aspects of dependency on cognitive technologies in general contexts.

### 6.2 Practical implications

These findings have several direct and concrete implications for university administration, curricula design, teachers, and policymakers. The basic lesson is that in addressing the psychological effects of dependence on AI systems, it is important to focus on the psychology underlying the problem, namely loss of sense of cognitive agency, rather than simply addressing the behavioral pattern of using AI systems. Limiting use of such tools would limit behavioral evidence of dependence, yet students with helplessness would carry their helplessness into AI-constrained contexts, suffering from motivational problems regardless of the presence of AI. Such a finding is supported by past work on the persistence of helplessness once acquired (Seligman, 1975; Abramson et al., 1978).

The most straightforward evidence-based intervention from this study targets AI literacy training. As suggested by the mediated moderation effects, students with greater AI literacy benefit from a significantly weakened indirect association between AI dependency and motivational harm. Training in AI literacy represents a direct psychological intervention within the path of helplessness, rather than being a mere digital skills training program. AI literacy interventions should focus on cultivating students' abilities to interpret and critique AI-generated material such that their perception of cognitive ownership remains intact. Instructors play an essential role in such endeavors by assigning activities in which students' cognitive contribution is transparent and evaluable, such as critical reflections on AI interactions or showcasing mastery in addition to completing AI-assisted applications. These measures do not call for the discontinuation of AI usage, but rather reframe students' cognitive interaction with AI in a manner that maintains agency rather than negates it.

At the institutional level, this study shows that AI literacy should be incorporated into the curriculum as a mandatory requirement for graduation, rather than being offered as an elective. The protective role played by AI literacy works regardless of the high dependence that learners have on AI, meaning that systematic investment in AI literacy development is likely to protect students' intrinsic motivation throughout their academic careers. The above suggestion is especially critical in the context of higher education in China, because of the academic competition that exists and the fast pace at which institutions adopt AI technology. Lastly, a validated learned helplessness test could act as an institution's early warning tool, enabling the university to detect students who are at risk of motivation loss (Liu et al., 2024; Wu et al., 2026).

Beyond these institutional recommendations, the study points toward specific and implementable changes at the classroom level. First, course design can integrate AI-embedded assessment tasks in which students are required to submit both an AI-assisted output and a written reflection documenting where AI was used, where AI's suggestions were rejected or revised, and what the student contributed independently. Such reflective assessments preserve visible cognitive authorship and interrupt the non-contingency perception that drives helplessness. Second, instructors can introduce structured AI usage guidelines at the start of each course that specify categories of appropriate use (e.g., brainstorming, formatting) and inappropriate use (e.g., generating final analytical arguments), thereby supporting the metacognitive awareness identified as central to AI literacy. Third, universities can offer short co-curricular AI literacy modules that combine technical instruction on how large language models function with practical exercises in evaluating AI-generated content for accuracy, bias, and completeness (Wang et al., 2022; Ng et al., 2021). These three practices, when adopted together, form a coherent educational response that preserves students' sense of cognitive agency without excluding AI from the learning environment.

## 7 Conclusion

This study set out to answer a question the field has left unanswered: through what psychological mechanism does AI dependency damage the intrinsic motivation of university students, and under what conditions can that damage be mitigated? Drawing on learned helplessness theory and self-determination theory, we proposed and tested a moderated mediation model in which learned helplessness transmits the negative effect of AI dependency on intrinsic motivation, with AI literacy serving as a boundary condition that attenuates the strength of this pathway. A sample of 457 students from Chinese universities was used in testing all five hypotheses, thus leading to the development of a consistent and theoretical explanation of the motivational processes brought about by AI dependency.

The core result shows that being dependent on AI does not negatively affect the intrinsic motivation either directly or because of decreased effort invested. Instead, it harms intrinsic motivation by creating a certain kind of psychological condition: a perception that one's own mental effort is not only unnecessary and inadequate but also irrelevant in terms of academic results. Such a condition is developed not because of failure but because of consistent successes that were attained through the process of cognitive displacement, which has never before been identified when applying LHT. With this condition created, students' autonomy and competence needs are hampered, and as a result, motivational issues emerge, just as educators have observed in the case of AI-dependent learners without understanding the exact psychology behind them.

Equally important is the finding that the process of psychological damage does not need to happen in the first place. In environments where students have a high degree of AI literacy, there will be no induction of learned helplessness even when they are highly dependent on AI. When AI literacy is understood as an attributional buffer rather than a technical skill, then it becomes the most effective tool available for safeguarding the motivational basis of education in the age of AI.

There are important limitations with this study. First, the cross-sectional design of this study does not allow causality to be determined. Whether the causality runs from learned helplessness to developing a reliance on AI, or whether the causality is reciprocal, can only be determined using other research methods in the future (Morales-García et al., 2024). One way to overcome this limitation is to conduct longitudinal research that studies the growth trajectory of learned helplessness, intrinsic motivation, and reliance on AI in a single semester (Galindo-Domínguez, 2025). Another approach would be to conduct experimental research in order to prove the causality between variables in question. The second important limitation of this study is its narrow sample—it included only Chinese university students. However, the academic environment of China's higher education system is distinguished by high performance pressures, collectivism in motivation orientations, and swift AI integration into institutions, all of which might mediate the hypothesized associations in different ways compared to Western or non-Chinese Asian countries (Zhong et al., 2024). The influence of AI dependence on learned helplessness will be more pronounced in the context of high academic performance pressures, while the role of AI literacy may differ due to various perceptions of AI legitimacy. Further studies need to explore the replicability and applicability of the above-presented model in other national and institutional contexts for defining the limits of its validity and cultural moderating factors ignored in the current study.

Third, the assessment of common method bias, although supplemented by procedural remedies and a common latent factor test, cannot fully rule out shared method variance given that all constructs were measured through student self-report on a single instrument. Future research should employ multi-source designs, such as combining student self-reports with teacher ratings or objective measures of AI use and academic performance, to strengthen the attribution of the observed relationships to substantive rather than methodological factors (Podsakoff et al., 2003).

Fourth, the external validity of the findings is constrained by the composition of the sample. Because participants were purposively drawn from Chinese universities where AI use is highly prevalent, the results may not generalize to institutions or student populations with lower AI adoption, different pedagogical traditions, or distinct cultural orientations toward technology use. Extending the model to comparative cross-national samples and to student groups with lower AI usage would clarify whether the observed AI dependency-helplessness-motivation pathway is a general phenomenon or specific to high-adoption contexts.

## Data Availability

The raw data supporting the conclusions of this article will be made available by the authors, without undue reservation.
