# Supplementary material for: AI dependency and academic motivation in higher education: the mediating role of learned helplessness and the moderating role of AI literacy
Source: Front Psychol. 2026 Jul 29;17:1896371. doi: 10.3389/fpsyg.2026.1896371 (PMC13462467; doi:10.3389/fpsyg.2026.1896371)
Supplement: Supplementary file 1 [file Supplementary_File_1.docx]

**Appendix A. Standardized Factor Loadings for All Items**

Confirmatory factor analysis yielded standardized loadings for all 45 items as follows. All loadings exceed 0.50 and are statistically significant at p < 0.001. Sample content is provided for illustration; the complete item wording follows the original validated scales cited in Section 4.2.

**AI Dependency – Cognitive Integration & Habit (Factor 1)**

| **Item** | **Sample content / description** | **Loading** |
| --- | --- | --- |
| AID_F1_1 | I feel AI has become an indispensable part of my knowledge system | 0.795 |
| AID_F1_2 | I habitually consult AI for academic tasks | 0.769 |
| AID_F1_3 | AI is central to how I complete assignments | 0.801 |
| AID_F1_4 | My knowledge feels incomplete without AI support | 0.836 |
| AID_F1_5 | I integrate AI outputs into most of my academic work | 0.766 |
| AID_F1_6 | AI is part of my routine for studying and learning | 0.766 |
| AID_F1_7 | I rely on AI for structuring my ideas | 0.838 |
| AID_F1_8 | AI has become my external cognitive resource | 0.806 |
| AID_F1_9 | My academic workflow depends heavily on AI | 0.756 |

**AI Dependency – Autonomy Erosion (Factor 2)**

| **Item** | **Sample content / description** | **Loading** |
| --- | --- | --- |
| AID_F2_1 | People around me say I cannot work or think without AI | 0.777 |
| AID_F2_2 | I feel anxious about performing academic tasks without AI | 0.736 |
| AID_F2_3 | I have difficulty starting an assignment without AI | 0.736 |
| AID_F2_4 | I no longer trust my judgment without AI verification | 0.765 |
| AID_F2_5 | My independent thinking has weakened due to AI use | 0.678 |
| AID_F2_6 | I feel dependent on AI even for simple academic decisions | 0.686 |

**Learned Helplessness (Chinese LHS)**

| **Item** | **Sample content / description** | **Loading** |
| --- | --- | --- |
| LH_1 | Learned helplessness item 1 | 0.730 |
| LH_2 | Learned helplessness item 2 | 0.709 |
| LH_3 | Learned helplessness item 3 | 0.769 |
| LH_4 | Learned helplessness item 4 | 0.714 |
| LH_5 | Learned helplessness item 5 | 0.691 |
| LH_6 | Learned helplessness item 6 | 0.821 |
| LH_7 | Learned helplessness item 7 | 0.745 |
| LH_8 | Learned helplessness item 8 | 0.758 |
| LH_9 | Learned helplessness item 9 | 0.691 |
| LH_10 | Learned helplessness item 10 | 0.731 |
| LH_11 | Learned helplessness item 11 | 0.760 |
| LH_12 | Learned helplessness item 12 | 0.703 |
| LH_13 | Learned helplessness item 13 | 0.772 |
| LH_14 | Learned helplessness item 14 | 0.728 |

**AI Literacy (Wang et al., 2022)**

| **Item** | **Sample content / description** | **Loading** |
| --- | --- | --- |
| AIL_1 | AI literacy item 1 | 0.732 |
| AIL_2 | AI literacy item 2 | 0.718 |
| AIL_3 | AI literacy item 3 | 0.828 |
| AIL_4 | AI literacy item 4 | 0.744 |
| AIL_5 | AI literacy item 5 | 0.697 |
| AIL_6 | AI literacy item 6 | 0.782 |
| AIL_7 | AI literacy item 7 | 0.690 |
| AIL_8 | AI literacy item 8 | 0.754 |
| AIL_9 | AI literacy item 9 | 0.657 |
| AIL_10 | AI literacy item 10 | 0.685 |
| AIL_11 | AI literacy item 11 | 0.754 |
| AIL_12 | AI literacy item 12 | 0.778 |

**Academic Intrinsic Motivation (AMS)**

| **Item** | **Sample content / description** | **Loading** |
| --- | --- | --- |
| IM_1 | I study because I experience pleasure in learning new things | 0.780 |
| IM_2 | I study for the satisfaction of mastering difficult material | 0.772 |
| IM_3 | I study because learning gives me joy and inspiration | 0.766 |
| IM_4 | I study to experience the pleasure of academic engagement | 0.731 |
